# Dissecting the genetic architecture of frost tolerance in Central European winter wheat

**DOI:** 10.1093/jxb/ert259

**Published:** 2013-09-04

**Authors:** Yusheng Zhao, Manje Gowda, Tobias Würschum, C. Friedrich H. Longin, Viktor Korzun, Sonja Kollers, Ralf Schachschneider, Jian Zeng, Rohan Fernando, Jorge Dubcovsky, Jochen C. Reif

**Affiliations:** ^1^Department of Cytogenetics and Genome Analysis, Leibniz Institute of Plant Genetics and Crop Plant Research (IPK), D-06466 Gatersleben, Germany; ^2^State Plant Breeding Institute, University of Hohenheim, D-70599 Stuttgart, Germany; ^3^KWS Lochow GmbH, D-29296 Bergen, Germany; ^4^Nordsaat Saatzuchtgesellschaft GmbH, D-38895 Langenstein, Germany; ^5^Department of Animal Science, Center for Integrated Animal Genomics, Iowa State University, Ames, IA 50011, USA; ^6^Department of Plant Sciences, University of California, Davis, CA 95616, USA; ^7^Howard Hughes Medical Institute, 4000 Jones Bridge Road, Chevy Chase, MD 20815, USA

**Keywords:** Association mapping, cross-validation, frost tolerance, genomic selection, *Triticum aestivum*, wheat.

## Abstract

Abiotic stress tolerance in plants is pivotal to increase yield stability, but its genetic basis is still poorly understood. To gain insight into the genetic architecture of frost tolerance, this work evaluated a large mapping population of 1739 wheat (*Triticum aestivum* L.) lines and hybrids adapted to Central Europe in field trials in Germany and fingerprinted the lines with a 9000 single-nucleotide polymorphism array. Additive effects prevailed over dominance effects. A two-dimensional genome scan revealed the presence of epistatic effects. Genome-wide association mapping in combination with a robust cross-validation strategy identified one frost tolerance locus with a major effect located on chromosome 5B. This locus was not in linkage disequilibrium with the known frost loci *Fr-B1* and *Fr-B2*. The use of the detected diagnostic markers on chromosome 5B, however, does not allow prediction of frost tolerance with high accuracy. Application of genome-wide selection approaches that take into account also loci with small effect sizes considerably improved prediction of the genetic variation of frost tolerance in wheat. The developed prediction model is valuable for improving frost tolerance because this trait displays a wide variation in occurrence across years and is therefore a difficult target for conventional phenotypic selection.

## Introduction

Wheat (*Triticum aestivum* L.) is one of the most important crops and is grown on 200 million hectares of farmland worldwide (Ortiz *et al.*, 2008). In the northern hemisphere, wheat is commonly planted in the autumn to escape the potential drought stress in the summer (Vágújfalvi *et al.*, 2012). Besides this, autumn-planted winter wheat possesses a higher yield potential than spring wheat owing to its longer growing period.

Frost tolerance is essential for autumn-planted wheat to survive freezing temperatures during winter in temperate zones (Sandve *et al.*, 2011). Therefore, the genetic architecture of frost tolerance has been investigated for almost a century (Skinner and Garland-Campbell, 2008; Galiba *et al.*, 2009). One prominent frost tolerance locus is *Fr-1* (Sutka and Snape, 1989; McIntosh *et al.*, 1998), which maps close to *Vrn-A1* and thus has been supposed to represent a pleiotropic effect of *Vrn-A1* (Dhillon *et al.*, 2010). An additional locus underlying frost tolerance, *Fr-A2*, has been identified on chromosome 5A ~30 cM away from *Vrn-A1* (Vágújfalvi *et al.*, 2000, 2003). Homoeologous loci were reported for chromosomes 5B (Toth *et al.*, 2003) and 5D (Snape *et al.*, 1997). In addition to the major *Fr-1* and *Fr-2* loci, there are multiple genes with small effects underlying frost tolerance. Therefore, while some progress can be achieved by selecting for beneficial *Fr-1 and Fr-2* alleles, once those are fixed in a breeding programme, additional tools are required to discover and deploy additional genes to further improve this trait.

Genomic selection has been suggested to predict phenotypes for traits that are controlled by multiple genes with small effects. In this approach, a large number of markers distributed across the genome are used simultaneously to train a prediction model (Meuwissen *et al.*, 2001). Genomic selection has shown great success in livestock improvement (Hayes, 2009) and more recently also in plant breeding (Jannink *et al.*, 2010). The simultaneous use of a large number of markers also has led to a substantially enhanced understanding of complex traits in human genetics (Lee *et al.*, 2008).

In this study, a large and diverse mapping population of 1604 wheat hybrids and their 135 parental inbred lines was evaluated for frost tolerance and fingerprinted with a 9K single-nucleotide polymorphism (SNP) array (Würschum *et al.*, 2013). Genome-wide association mapping was applied to study the genetic architecture of frost tolerance in winter wheat, and one major additive effect quantitative trait locus (QTL) was revealed on chromosome 5B. The scan for dominance effects determined their contribution to genetic variation, which was, however, considerably smaller than that of the additive effects. Similarly, several interaction effects were identified among genes, each explaining only a small proportion of the genetic variation of frost tolerance. In contrast to the genome-wide association mapping, the application of two genomic selection approaches that take into account also loci exhibiting small size effects led to a considerable increase in the accuracy of frost tolerance prediction in wheat.

## Materials and methods

### Plant material and field experiments

This study was based on 1604 elite wheat hybrids and their 135 parental inbred lines adapted to Central Europe (Supplementary Table S1, available at *JXB* online). For hybrid seed production, the 135 parental inbred lines were classified into a male group consisting of tall and open-pollinating genotypes and a female group of semi-dwarf genotypes showing a short delay in flowering time compared to the male lines. The hybrids were derived by crossing 120 female and 15 male lines (Supplementary Fig. S1) using chemical hybridization agents. The 1739 genotypes were evaluated for frost tolerance in field trials at three locations in Germany (Seligenstadt: 50° 2′ N 8° 58′ E, 278 m above sea level, silt clay loam soil texture; Böhnshausen: 51° 51′ N 10° 57′ E, 146 m above sea level, sandy loam texture; Adenstedt: 52° 0′ N 9° 56′ E, 71 m above sea level, loam soil texture) in the year 2012. The experimental designs were partially replicated alpha designs and 29% of the hybrids were tested in two replications (Williams *et al.*, 2010). The hybrids and lines were split into three trials linked with 10 common checks (*Asdecouer*, *Genius*, *Hystar*, *JBAsano*, *Julius*, *Tuerkis*, *Colonia*, *Kredo*, *Tobak*, and *Tabasco*). Sowing was done in the first 2 weeks of October and sowing densities ranged from 230 to 290 grains m^–2^ and plot sizes ranged from 7.5 to 9.7 m^2^. The plots were treated with fertilizers, fungicides, and herbicides according to standard agronomic practices for intensive wheat production. Frost tolerance was visually scored on a scale from 1 (no damage) to 9 (no plant survived).

### Genotypic data

Genotyping was done with the 9K SNP array based on the Illumina Infinium assay (for details see Würschum *et al.*, 2013). Quality checks for the SNP markers were performed to exclude those showing: (1) rate of missing values above 5%; (2) rate of heterozygosity in the parental inbred lines above 5%; or (3) minor allele frequency smaller than 0.05. In total, 1280 SNP markers were retained and missing genotypes were imputed following the approach suggested by Crossa *et al.* (2010; Supplementary Table S2). This work followed the suggestion of Bernardo (1993) and estimated the coancestry coefficients *θ*_*ij*_ between inbreds *i* and *j* on the basis of marker data as *θ*_*ij*_ = 1 + (*S*_*ij*_ – 1)/(1 – *T*), where *S*_*ij*_ is the proportion of marker loci with shared variants between inbreds *i* and *j* and *T* is the average probability that a variant from one parent of inbred *i* and a variant from one parent of inbred *j* are alike in state, given that they are not identical by descent. *T* was set as minimum of (1 – *S*_*ij*_) values.

### Phenotypic data analyses

The phenotypic data of each environment were first analysed separately based on the statistical model


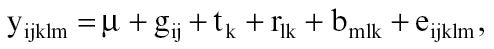


where *y*_*ijklm*_ was the phenotypic performance for the *ij*th genotype in the *m*th incomplete block of the *l*th replication in the *k*th trial, *µ* was an intercept term, *g*_*ij*_ was the genetic effect of the *ij*th genotype, *t*_*k*_ was the effect of the *k*th trial, *r*_*lk*_ was the effect of the *l*th replication in the *k*th trial, *b*_*mlk*_ was the effect of the *m*th incomplete block in the *l*th replication of the *k*th trial, and *e*_*ijklm*_ was the residual. Except *b*_*mlk*_, all effects were treated as fixed. Adjusted entry means were used in a second step to estimate the genetic variance components of hybrids and parental lines as well as the variance of genotype × location interactions. This work followed the suggestion of Möhring and Piepho (2009) and weighted each observation with one divided by their squared standard error. The variance of hybrids was further split into variance due to general and specific combining ability effects (Hallauer and Miranda, 1988). Significance of variance component estimates was tested by model comparison with likelihood ratio tests in which halved *P*-values were used as approximation (Stram and Lee, 1994). In addition, this work assumed fixed genetic effects and obtained the best linear unbiased estimates of the 1739 genotypes. The phenotypic data analyses were performed using the software ASReml 3.0 (Gilmour *et al.*, 2009).

### Genome-wide mapping

The additive and dominance design matrices for the hybrids and their parental lines were specified according to the F_∞_ metric of Falconer and Mackay (1996). Based on the adjusted entry means of the single locations, association mapping scans were performed for additive and dominance effects correcting for population stratification by fitting a polygenic effect for each individual as random, where the covariance for the polygenic effect is the kinship matrix estimated from the marker data (Yu *et al.*, 2006). The kinship matrix for the parental lines was modelled using twice the estimated coancestry coefficients *θ*_*ij*_ between inbreds *i* and *j* on the basis of marker data (Reif *et al.*, 2011). The general combining ability effects reflect the additive effects of the hybrids (Hallauer and Miranda, 1988). Therefore, the kinship matrix for the hybrids modelled the covariance among general combining ability effects (Reif *et al.*, 2013). This approach was contrasted with a Null model not correcting for population stratification and two models fitting an average heterotic effect (contrast of lines versus hybrids) considering and disregarding the kinship matrix. The significance of genome-wide association mapping scans for the main effects was estimated based on a false discovery rate (Benjamini and Hochberg, 1995) of 0.1. In addition, this work extended the tests for main effect and performed a two-dimensional genome scan for additive × additive, additive × dominance, dominance × additive, and dominance × dominance digenic epistatic effects. The extended biometrical model included the detected main-effect quantitative trait loci (QTL) as co-factors as well as the main and interaction effects of the marker pair under consideration (Würschum *et al.*, 2011). The Bonferroni–Holm procedure (Holm, 1979) was applied to correct for multiple testing. The association mapping analyses were performed using the software ASReml 3.0 (Gilmour *et al.*, 2009).

Based on the adjusted entry means of the 1739 genotypes, two approaches for genomic selection were applied considering additive and dominance effects: ridge regression best linear unbiased prediction (RR-BLUP; Whittaker *et al.*, 2000) and BayesCπ (Dekkers *et al.*, 2009; Habier *et al.*, 2011). Details of the implementation of the models have been described in Zhao *et al.* (2013). All statistical procedures for the genomic selection approaches were executed using R (R Development Core Team, 2010).

The accuracy of the prediction of frost tolerance by association mapping and the two genomic selection approaches were evaluated using cross-validations. Since population structure in factorial crosses strongly influences prediction accuracy (Technow *et al.*, 2012), a cross-validation strategy was used where training and validation sets were not related by common parental lines. This work sampled 100 times 610 hybrids and their 10 male and 80 female parental lines as the training set and estimated the additive and dominance effects. Hybrids based on the remaining parental lines formed the validation set in which predictions derived from the training set were tested for their accuracy. Prediction accuracy was estimated as Pearson’s correlation coefficient between the observed and the predicted hybrid performance.

## Results

The 1739 entries were evaluated for frost tolerance occurring in the field under natural settings. Frost stress was severe at all three locations in the first half of February 2012, with minimum temperature down to –18 °C (Supplementary Fig. S2) concomitant with an absence of snow coverage and soil-borne diseases. The high correlations observed among the genotypic values estimated in the three locations (Supplementary Table S3) clearly suggested that a combined analysis across locations would not be biased by location-specific frost responses. The high frost stress revealed a large genetic variation for frost tolerance in the mapping population with high heritabilities (close to 90%; Table 1). The exceptional differentiation obtained under the natural setting is also underlined by the large difference observed in frost tolerance between the check varieties *Julius* and *Tabasco* (Supplementary Fig. S3). *Julius* is known to be frost tolerant and *Tabasco* frost susceptible.

**Table 1.** First- and second-degree statistics for the 1604 hybrids and their 135 parental inbred lines evaluated for frost tolerance in field trials at three locations

Source of variation	Frost tolerance
Lines	5.51 (1.40–8.94)
Hybrids	4.43 (1.28–8.58)
σ^2^_Lines_	3.14***
σ^2^_Hybrids_	1.66***
σ^2^_Additive-Female_	1.51***
σ^2^_Additive-Male_	0.26***
σ^2^_Dominance_	0.08***
σ^2^_Lines × Location_	0.73***
σ^2^_Hybrids × Location_	0.38***
σ^2^_Additive-Male × Location_	0.36***
σ^2^_Additive-Female × Location_	0.07***
σ^2^_Dominance × Location_	0.04***
σ^2^_e_	0.52
*Heritability*_*Lines*_	0.89
*Heritability*_*Hybrids*_	0.87

Values are mean (range) or variances. Frost tolerance was scored from 1 (no damage) to 9 (no plant survived).

*** Significantly different from zero at *P* ≤ 0.001.

This study observed a negligible correlation of 0.02 between frost tolerance and heading time. This indicated that the observed genotypic variation for frost tolerance was not likely to have been caused by differences in the transition from the vegetative to the generative growth phase. Nevertheless, as the transition from vegetative to generative growth was not measured directly, possible effects of the transition on frost tolerance cannot be ruled out completely.

Based on the genome-wide SNP marker data, there was no major population structure, but the presence of a family structure among the 135 parental lines (Supplementary Fig. S4). This can be explained by a steady interchange of lines among different wheat breeding programmes in their breeding history (Longin *et al.*, 2012). The quantile–quantile plots for the four association mapping models revealed that, owing to the presence of family structures (Supplementary Fig. S4), population stratification had to be considered through the kinship matrix for the lines and hybrids for a single-locus regression model (Supplementary Fig. S5). Fitting an average heterotic effect, however, was not required. Mapping QTL in a combined population of hybrids and lines led to a substantially higher proportion of genotypic variance explained by the QTL (Supplementary Fig. S5). This held true even if the proportion of genotypic variation explained by SNPs was estimated separately for the parental lines and the hybrids. Therefore, the genome-wide mapping approaches were based on the combined population of hybrids and lines.

**Fig. 1.**
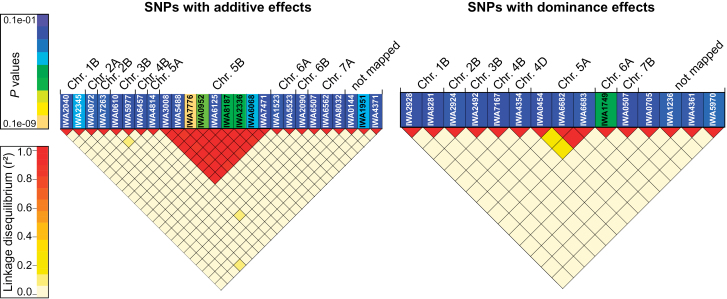
Heatplots of *P*-values of SNP markers contributing significantly to the additive and dominance genetic variation and linkage disequilibrium measured as squared Pearson’s correlation coefficients (*r* ^2^) among SNPs.

In the genome-wide association mapping scan, there were 26 and 15 SNPs which contributed significantly to the additive and dominance genetic variation for frost tolerance, respectively (Fig. 1). About 75% of these 41 SNPs exhibited significant (*P* < 0.01) interaction effects with the environments. A full two-dimensional scan for epistatic effects was performed, which revealed a total of 76 significant digenic epistatic effects. The distribution of the *P*-values revealed that, among the four types of digenic epistatic effects, additive × additive interactions were the most prevailing (Supplementary Fig. S6).

**Fig. 2.**
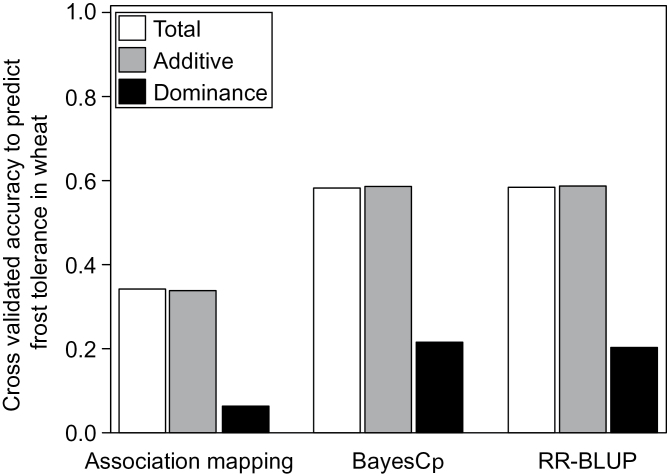
Cross-validated accuracy to predict frost tolerance in wheat based on association mapping and the two genomic selection methods BayesCπ and RR-BLUP.

Using a robust cross-validation strategy in which training and validation sets were not linked through common parents, the phenotypic performance of unrelated hybrids could be predicted with an accuracy of 0.34 based on the SNPs detected in the association mapping scans (Fig. 2). By standardizing this with the square root of the broad-sense heritability (Dekkers, 2007), the accuracy increased up to 0.38. Employing only five linked SNPs that were significantly associated with frost tolerance in more than 90% of the cross-validation runs (Supplementary Table S4) was sufficient to explain 98% of the prediction accuracy of the total set of significant SNPs. These five SNPs all exhibit only significant additive effects, are located in the same genomic region on chromosome 5B, and have *r* ^2^ values amongst each other above 0.88.

The location of these five SNPs suggests that this locus is proximal to *Fr-B1/Vrn-B1*, which is located roughly in the middle of the long arm (~70 cM from the centromere; Dubcovsky *et al.*, 1998). By using previously developed PCR markers for this locus (Fu *et al.*, 2005), this work confirmed that they are not linked to *Fr-B1/Vrn-B1*. Pearce *et al.* (2013) reported recently that a substantial proportion of the phenotypic variation of frost tolerance among spring wheats was associated with deletions involving *CBF* (*C-repeat Binding Factor*) genes at the *Fr-B2* locus, also located on the long arm of chromosome 5B. The current work used the developed PCR markers for *CBF-B9* and *CBF-B14* (Pearce *et al.*, 2013), which were segregating in the 135 parental wheat lines, and observed the absence of linkage disequilibrium between the alleles of the SNPs detected in the scan on chromosome 5B and the polymorphisms at the *Fr-B2* locus with *r* ^2^ values smaller than 0.02.

The genomic selection approaches led to an accuracy of predicting the phenotypic performance of unrelated hybrids of 0.58, a substantial increase (nearly 60%) compared to the association mapping approach. BayesCπ and RR-BLUP showed negligible differences in the accuracy to predict frost tolerance despite the low posterior inclusion of markers in BayesCπ (20% additive and 6% dominance). There was an accuracy of prediction of frost tolerance with dominance effects based on RR-BLUP and BayesCπ of around 0.20. Nevertheless, a combined prediction considering additive and dominance effects was not superior to predicting frost tolerance exclusively by additive effects.

## Discussion

A crucial factor in determining the quality of molecular data for genome-wide mapping studies is the extent of linkage disequilibrium. Theoretical findings (Hayes *et al.*, 2009) suggest that a SNP marker density resulting in mean *r* ^2^ values among adjacent loci of above 0.2 is useful for genome-wide approaches to predict phenotypic performance. The current study’s companion paper (Würschum *et al.*, 2013) estimated that the marker density underlying this study provided a magnitude of linkage disequilibrium measured as mean squared Pearson’s correlation coefficient (*r* ^2^) between adjacent SNPs of 0.32, which suggests that genome-wide mapping is promising with this experimental set up.

The genome-wide association mapping scan in combination with the robust cross-validation approach suggests that one gene with a major effect is present roughly in the middle of the long arm of chromosome 5B (Fig. 1). Interestingly, among the SNPs in that region, there were large differences in *P*-values, ranging from 4.4E–4 for IWA6125 to 8.5E–14 for IWA7776. This is surprising because this study observed high *r* ^2^ (above 0.9) between adjacent SNPs. Obviously the mapping resolution can still profit from the 10% of variation not explained by adjacent SNPs. The alleles of the SNPs detected in the scan on chromosome 5B were not in linkage disequilibrium with known polymorphisms at the *Fr-B1* and *Fr-B2* loci. Consequently, these findings suggested that a new frost stress locus had been detected on chromosome 5B.

Previous studies suggested that *Vrn-A1* contributes substantially to the genetic variation of frost tolerance when differences are tested between winter and spring alleles (Sutka and Snape, 1989). However, significant effects associated to *Vrn-A1* have been also detected among winter wheat varieties (Chen *et al.*, 2009). One out of the 1280 SNPs (IWA0454) was tightly linked with two previously described SNPs in the *Vrn-A1* gene (Chen *et al.*, 2009; Díaz *et al.*, 2012). Adding the marker IWA0454 in the cross-validation study resulted in an increase of 10% in accuracy compared to predicting frost tolerance based on the five SNPs described above. This underlines the possibility that *VRN-A1* is involved in the determination of frost tolerance of elite wheat varieties, although with lower impact as expected from previous studies (Dhillon *et al.*, 2010). The *Fr-2* locus (Vágújfalvi *et al.*, 2000) explained up to 40% of the phenotypic variation of frost tolerance in bi-parental QTL mapping studies based on contrasting parents (e.g. Båga *et al.*, 2007). However, no major effect loci were observed on chromosome 5A. This can be explained either by fixation of the *Fr-A2* locus in the lines adapted to Central Europe, a strong dependency of estimated marker effects on the genetic background (Miedaner *et al.*, 2011), or by the lack of SNPs in linkage disequilibrium with the causal alleles of the *Fr-A2* locus.

The association mapping scan yielded stable diagnostic markers only for one genomic region with a restricted potential to predict frost tolerance of wheat genotypes. This can be explained by a low number of large-effect QTLs underlying frost tolerance and the low power of association mapping strategies to tackle QTL with small to medium effect sizes. To overcome this limitation, two genomic selection approaches were used. While in BayesCπ only a fraction of 1 – π SNPs is assumed to contribute to frost tolerance, RR-BLUP assumes that all markers contribute to the trait. The accuracy in predicting frost tolerance was nearly 60% higher with BayesCπ (Fig. 2) as compared with association mapping. Frost tolerance was influenced by a subset of approximately 265 SNPs with additive effects and 74 SNPs with dominance effects. However, it is known from simulation studies (Habier *et al.*, 2011) that these numbers tend to be overestimated. Assuming an infinitesimal model and applying RR-BLUP yielded similar accuracies to predict frost tolerance. This is surprising since BayesCπ is expected to tackle traits with a mixture of large-, medium-, and small-effect QTLs more appropriately than RR-BLUP (Daetwyler *et al.*, 2010). A closer examination of the marker effects estimated for RR-BLUP, however, revealed that the large-effect QTL on chromosome 5B, for example, is captured through a number of linked SNPs with a sum of absolute values of the additive effects (0.55) comparable to BayesCπ (0.89).

**Fig. 3.**
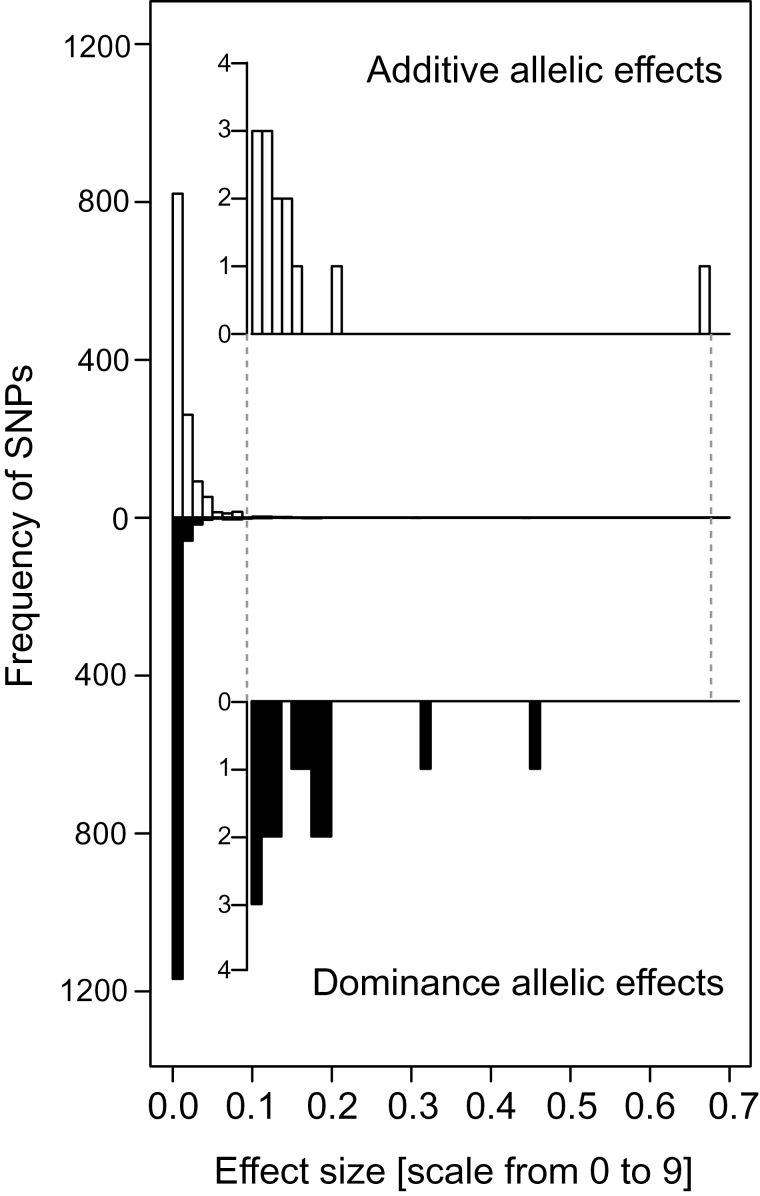
Distribution of marker effects estimated using BayesCπ for frost tolerance measured from a scale from 1 (no damage) to 9 (no plant survived).

Across all mapping approaches, the accuracy of prediction of frost tolerance by dominance effects alone was smaller compared to the prediction exploiting exclusively additive effects (Fig. 2). This can be explained by the lower contribution of dominance effects to the genetic variation (Table 1; Figure 3), as expected for the autogamous crop wheat (Longin *et al.*, 2012). In contrast to previous studies (Lee *et al.*, 2008; Wittenburg *et al.*, 2011), combining additive and dominance effects did not lead to higher accuracies of prediction of frost tolerance (Fig. 2). This seemed surprising as dominance captures a new source of variation, but can be explained by the large proportion of unexplained variation entering the prediction model when dominance effects are included (Zhao *et al.*, 2013).

The observed accuracy of prediction of frost tolerance (Fig. 2) suggests that there is still room for improvement. One possible approach to increase the ability to predict frost tolerance in wheat is to further enhance the marker density, for example by employing genotyping-by-sequencing approaches. This can be important to tackle the underlying causal polymorphisms with a higher precision compared to the use of a 9K SNP array. The assumption of an absence of epistasis is another potential reason for the still imperfect accuracy of prediction of frost tolerance (Zuk *et al.*, 2012), as supported by previous studies on epistatic interactions among key players underlying frost tolerance (Galiba *et al.*, 2009). To study this in more detail, the current work extended the main-effect association mapping approach towards a full two-dimensional scan for interaction-effects among SNPs. Among the four types of digenic epistatic effects, additive × additive effects were the most prevailing ones in terms of effect size (Supplementary Fig. S7) and skewness of *P*-values towards zero (Supplementary Fig. S6). This predominance of additive × additive effects is expected for selfing species such as wheat (Cockerham, 1984). The identified epistatic effects were, however, rather small, with a maximum contribution of a single effect to the phenotypic variation of 1.7% (Supplementary Fig. S7). Despite these small effect sizes, the large number of potential interaction effects can lead to a substantial contribution of epistasis to the phenotypic variation of frost tolerance. Consequently, the presence of epistatic effects is an explanation for this study’s inability to predict frost tolerance with a higher accuracy than observed.

This study provides insights into the genetic architecture of frost tolerance in wheat adapted to Central Europe. One major QTL on chromosome 5B segregating in European winter wheat was detected, which is of use for marker-assisted selection after thorough validation in independent experiments. In addition, for the first time it has been shown that using genomic selection approaches that assume a more complex genetic architecture represents a major step to improve understanding of the genetic architecture of frost tolerance. The developed calibration models are valuable to improve the frost tolerance of wheat, which is a trait that displays a wide variation in occurrence across years and is therefore a difficult target for conventional phenotypic selection.

## Supplementary material

Supplementary data are available at *JXB* online.

Supplementary Fig. S1. Crossing scheme between the 120 female and 15 male parental wheat lines

Supplementary Fig. S2. Temperature profiles of the three locations

Supplementary Fig. S3. Distribution of the phenotypic values of 1739 genotypes for frost tolerance

Supplementary Fig. S4. Coefficients of parentage estimated for the 120 female and 15 male parental wheat lines from SNP marker data

Supplementary Fig. S5. Quantile–quantile plots for association mapping based on the combined population of hybrids and parental lines using four different biometrical approaches

Supplementary Fig. S6. Distribution of *P*-values for four different types of digenic epistatic effects

Supplementary Fig. S7. Box-whisker plots of the proportion of explained phenotypic variation for significant digenic epistatic effects

Supplementary Table S1. Description of the 135 parental lines used in this study

Supplementary Table S2. SNP data of the 135 parental lines

Supplementary Table S3. Phenotypic correlations of the adjusted entry means of 1604 hybrids and 135 lines of three environments

Supplementary Table S4. Summary of marker statistics for the three applied genome-wide mapping approaches

Supplementary Data
