# Supplementary material for: Dissecting the genetic architecture of frost tolerance in Central European winter wheat
Source: J Exp Bot. 2013 Sep 4;64(14):4453–60. doi: 10.1093/jxb/ert259 (PMC3808325; doi:10.1093/jxb/ert259)
Supplement: Supplementary Data [file supp_64_14_4453__index.html]

Dissecting the genetic architecture of frost tolerance in Central European winter wheat — Supplementary Data 

# Dissecting the genetic architecture of frost tolerance in Central European winter wheat

## Supplementary Data

Data files

**Files in this Data Supplement:**

- Supplementary Data - Supplementary Data
- Supplementary Data - Supplementary Data
