# Supplementary material for: Dissecting the genetic architecture of frost tolerance in Central European winter wheat
Source: J Exp Bot. 2013 Sep 4;64(14):4453–60. doi: 10.1093/jxb/ert259 (PMC3808325; doi:10.1093/jxb/ert259)
Supplement: Supplementary Data [file supp_ert259_jexbot099374_file002.pdf]

Dissecting the genetic architecture of frost tolerance in Central European winter wheat

Yusheng Zhao, Manje Gowda, Tobias Würschum, Friedrich Longin, Viktor Korzun, Sonja Kollers, Ralf Schachschneider, Jian Zeng, Rohan Fernando, Jorge Dubcovsky, and Jochen Reif

Supplementary Data

Supplementary Tables S1-S4 – see separate file

Supplementary Figures S1-S7- below

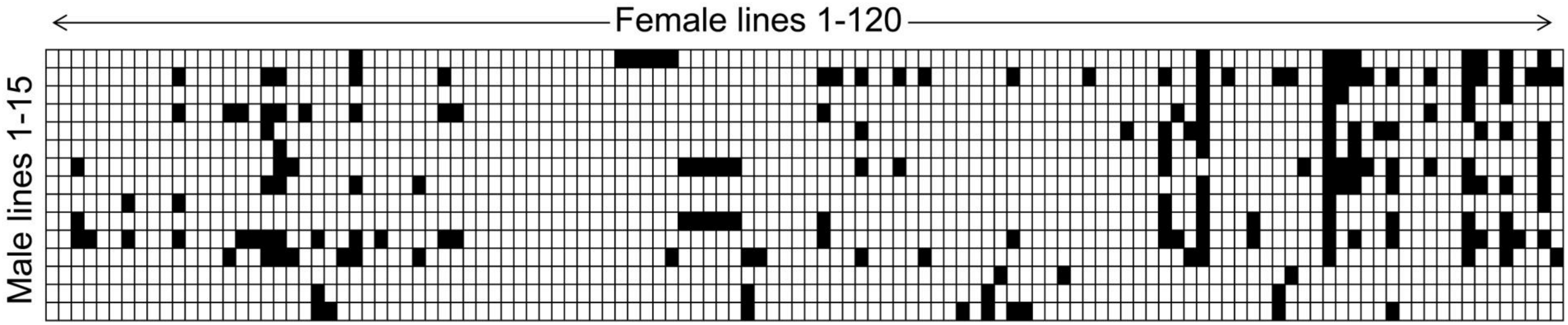

Supplementary Fig. S1. Crossing scheme between the 120 female and 15 male parental wheat lines. Non-filled boxes indicate presence and filled boxes indicate absence of a particular cross among hybrids included in analysis.

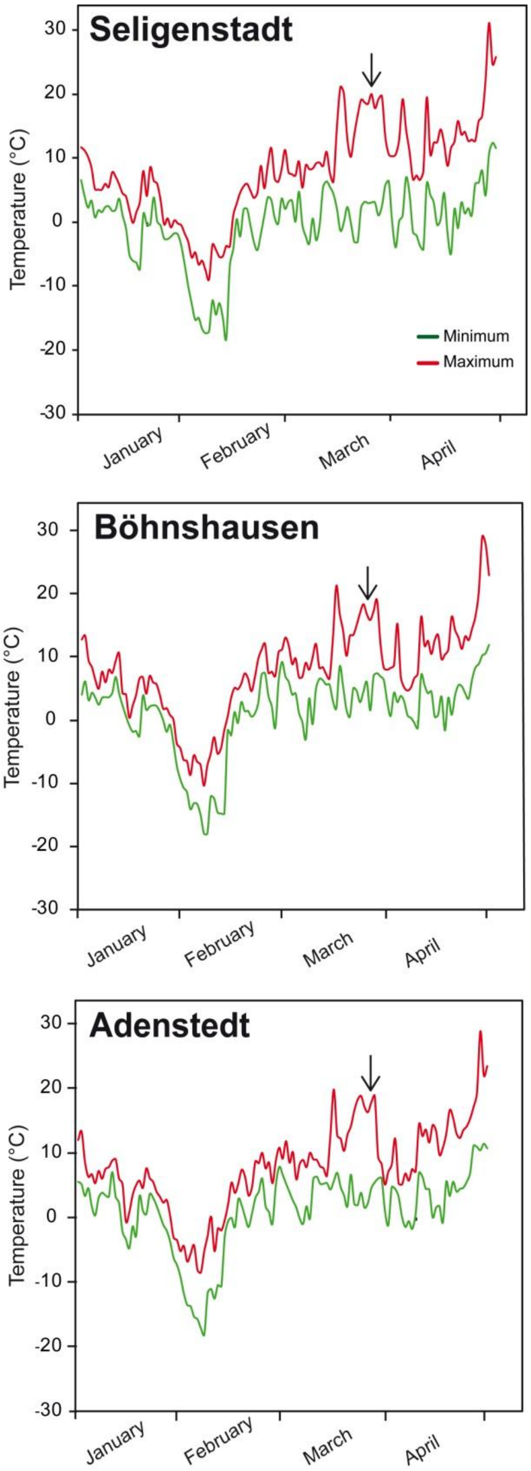

Supplementary Fig. S2. Temperature profiles of the three locations. Arrows indicate the date of phenotyping.

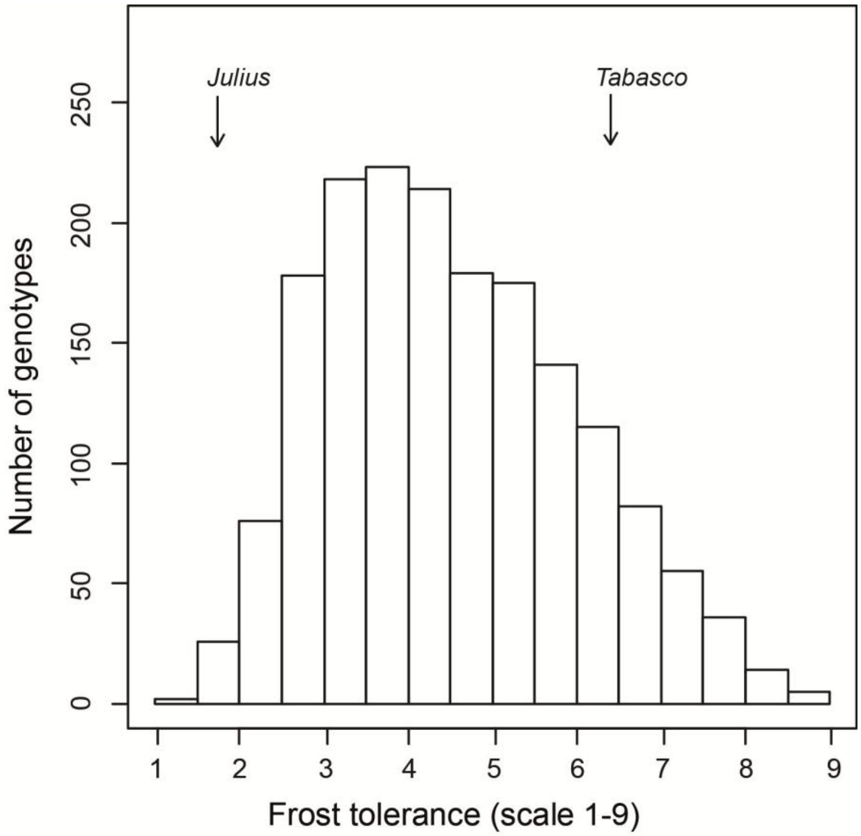

Supplementary Fig. S3. Distribution of the phenotypic values of 1739 genotypes for frost tolerance

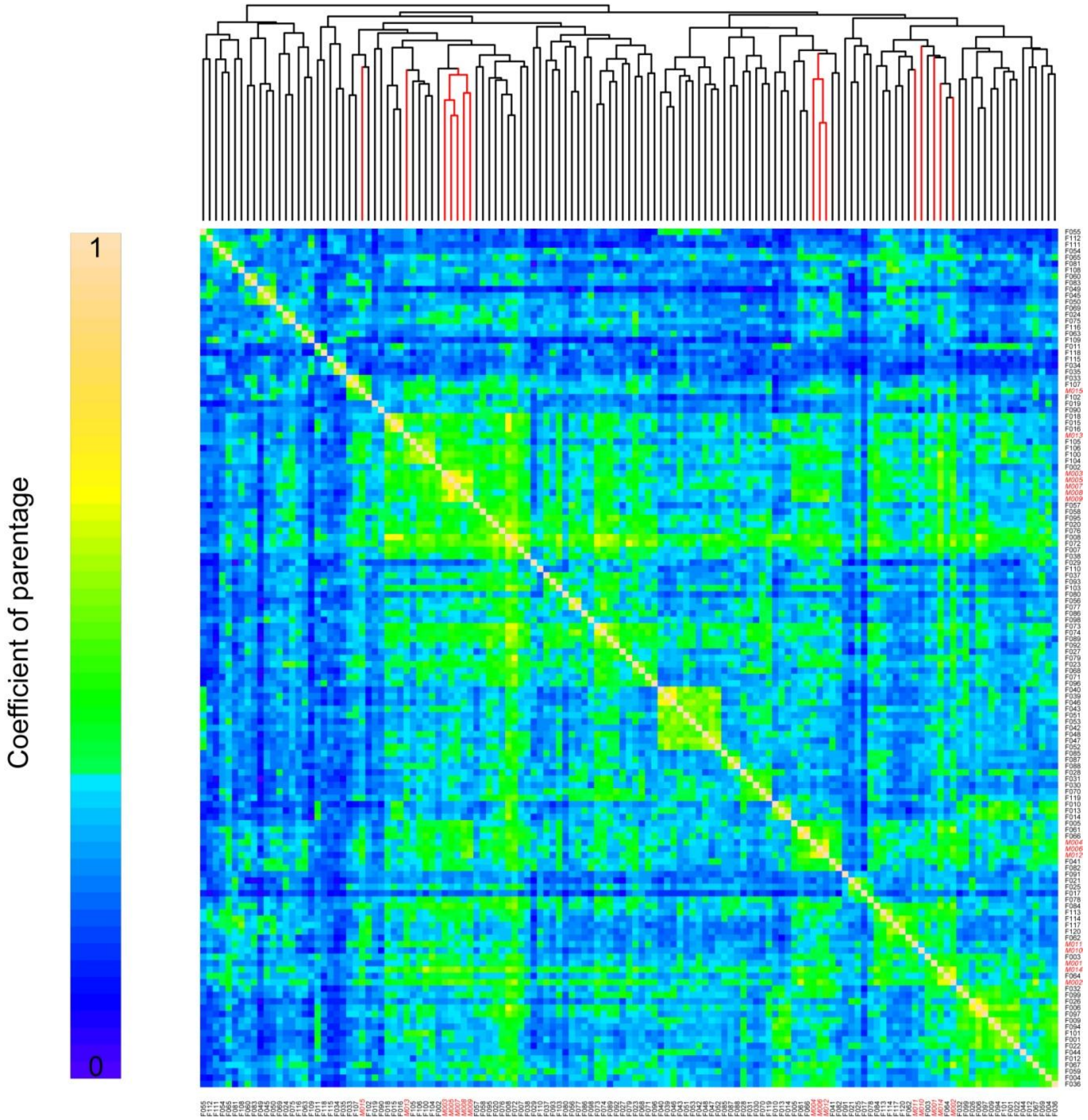

Supplementary Fig. S4. Coefficients of parentage estimated for the 120 female and 15 male parental wheat lines from SNP marker data. Average linkage clustering was used for ordering the individuals. Male lines are marked in red and female lines in black.

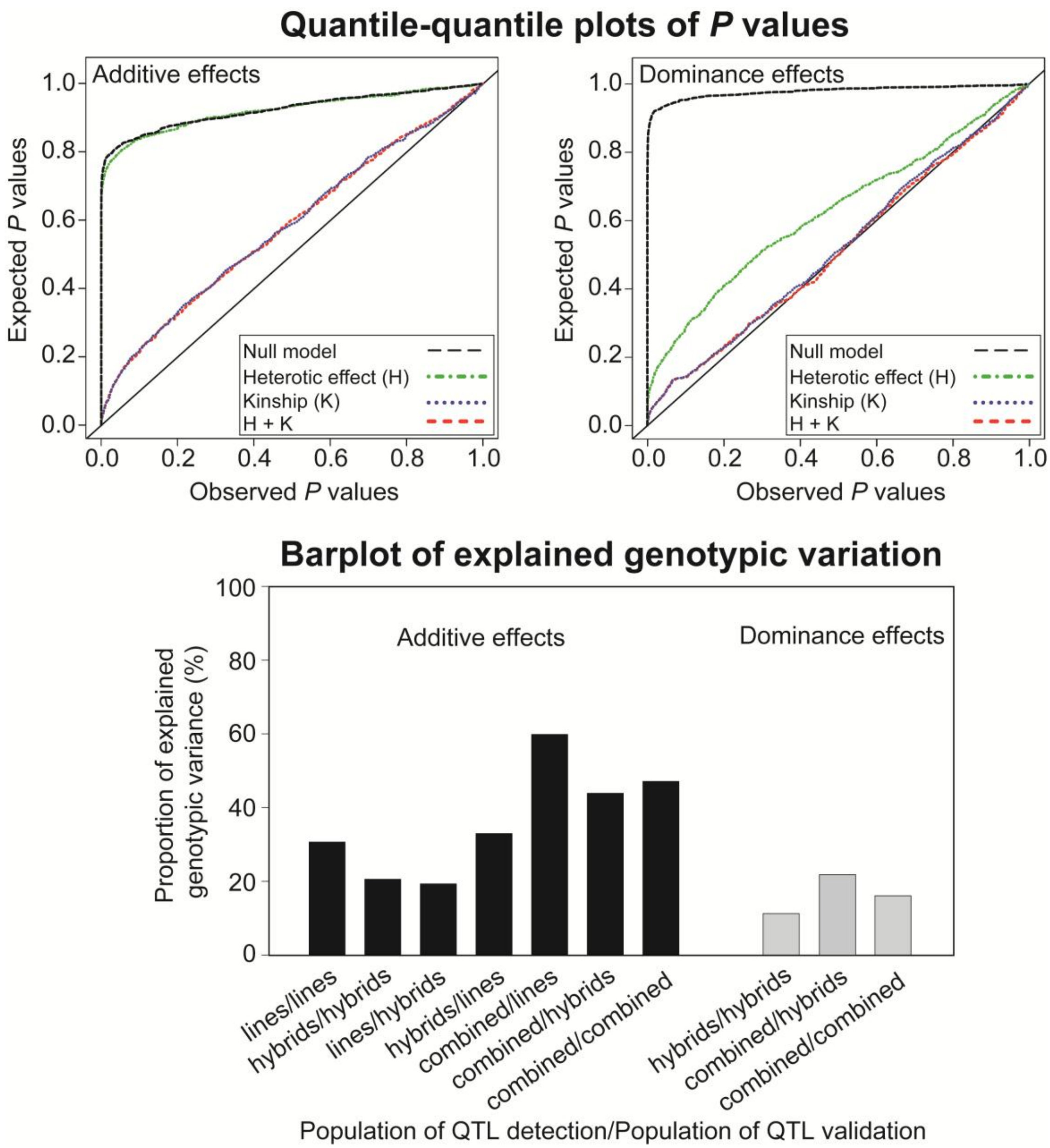

Supplementary Fig. S5. Quantile-quantile plots for association mapping based on the combined population of hybrids and parental lines using four different biometrical approaches: (1) without correction for population structure, (2) correcting for population structure with a heterotic effect, (3) correcting for population structure with a kinship matrix, and (4) correcting for population structure with a kinship matrix and a heterotic effect. The bar diagram shows the explained proportion of the genotypic variance of QTL detected and validated in different populations.

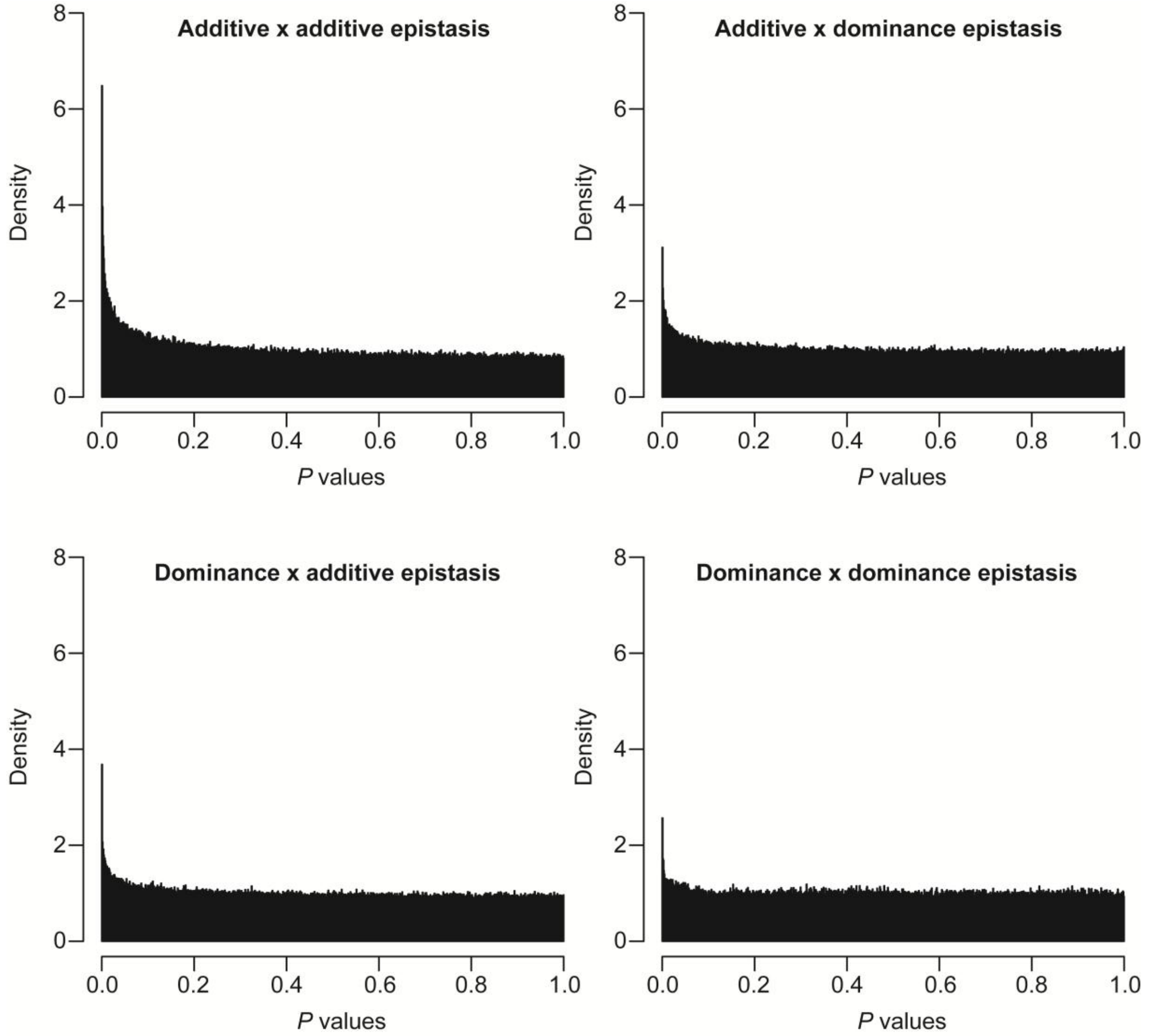

Supplementary Fig. S6. Distribution of *P* values for 4 different types of digenic epistatic effects.

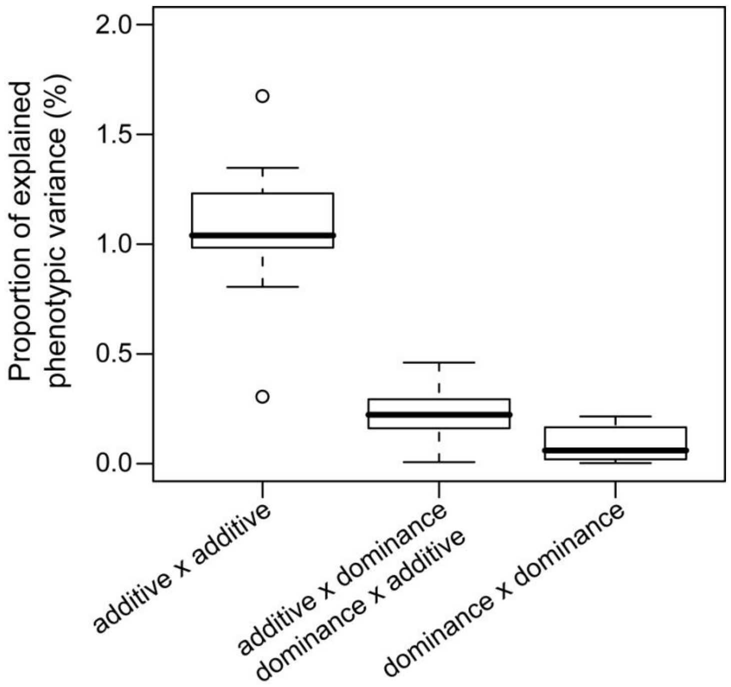

Supplementary Fig. S7. Box-Whisker plots of the proportion of explained phenotypic variation for significant digenic epistatic effects.
